# Postoperative chemoradiation for resected gastric cancer - is the Macdonald Regimen Tolerable? a retrospective multi-institutional study

**DOI:** 10.1186/1748-717X-6-127

**Published:** 2011-09-29

**Authors:** Yulia Kundel, Ofer Purim, Efraim Idelevich, Konstantin Lavrenkov, Sofia Man, Svetlana Kovel, Natalia Karminsky, Raphael M Pfeffer, Bella Nisenbaum, Eyal Fenig, Aaron Sulkes, Baruch Brenner

**Affiliations:** 1 Davidoff Cancer Center, Rabin Medical Center, Beilinson Campus and Sackler Faculty of Medicine, Tel Aviv University, Tel Aviv, Israel; 2 Institute of Oncology, Kaplan Medical Center, Israel; 3 Department of Oncology, Soroka University Medical Center, Israel; 4 Institute of Oncology, Asaf Harofeh Medical Center, Israel; 5 Institute of Oncology, Wolfson Medical Center, Israel; 6 Institute of Oncology, Chaim Sheba Medical Center and Sackler Faculty of Medicine, Tel Aviv University, Tel Aviv, Israel; 7 Institute of Oncology, Meir Medical Center, Israel

**Keywords:** Postoperative chemoradiation, resected gastric cancer, Israeli experience

## Abstract

**Background:**

Postoperative chemoradiation as per Intergroup-0116 trial ("Macdonald regimen") is considered standard for completely resected high risk gastric cancer. However, many concerns remain with regards to the toxicity of this regimen. To evaluate the safety and tolerability of this regimen in a routine clinical practice setting, we analyzed our experience with its use. As we did not expect a different toxic profile in patients (pts) with positive margins (R1 resection), these were studied together with pts after complete resection (R0).

**Patients and Methods:**

Postoperative chemoradiation therapy was given according to the original Intergroup-0116 regimen. Overall survival (OS) and disease free survival (DFS) rates were calculated using the Kaplan-Meier method. Comparison of OS and DFS between R0 and R1 pts was done using the log-rank test.

**Results:**

Between 6/2000 and 12/2007, 166 pts after R0 (129 pts) or R1 (37 pts) resection of locally advanced gastric adenocarcinoma received postoperative chemoradiation; 61% were male and the median age was 63 years (range, 23-86); 78% had T ≥ 3 tumors and 81% had N+ disease; 87% of the pts completed radiotherapy and 54% completed the entire chemoradiation plan; 46.4% had grade ≥ 3 toxicity and 32% were hospitalized at least once for toxicity. Three pts (1.8%) died of toxicity: diarrhea (1), neutropenic sepsis (1) and neutropenic sepsis complicated by small bowel gangrene (1). The most common hematological toxicity was neutropenia, grade ≥ 3 in 30% of pts and complicated by fever in 15%. The most common non-hematological toxicities were nausea, vomiting and diarrhea. With a median follow-up of 51 months (range, 2-100), 62% of the R0 patients remain alive and 61% are free of disease. Median DFS and OS for R0 were not reached. R0 pts had a significantly higher 3-year DFS (60% vs. 29%, p = 0.001) and OS (61% vs. 33%, p = 0.01) compared with R1 pts.

**Conclusions:**

In our experience, postoperative chemoradiation as per Intergroup-0116 seems to be substantially toxic, with a mortality rate which seems higher than reported in that trial. Efficacy data appears comparable to the original report. Following postoperative chemoradiation, involvement of surgical margins still has a detrimental impact on patient outcome.

## Introduction

Gastric cancer is the second leading cause of cancer related death among men and the fourth among women, and thus represents a significant global health concern [1]. The disease is commonly diagnosed at an advanced stage, either with extensive locoregional involvement or with overt distant metastases. Overall 5-year survival rate approximates 20% and has undergone minimal change over the last decade [1].

Complete surgical resection of gastric cancer is curative in less than 40% of cases [2]. In patients with deep invasion of the gastric wall or regional lymph node metastases the relapse and death rates from recurrent cancer exceed 70-80%. Loco-regional recurrences in the tumor bed, the anastomosis or in regional lymph nodes occur in 40 to 65% of patients after curative intent resection [3]; the frequency of this relapse makes regional radiotherapy an attractive possibility for adjuvant therapy.

Most previous adjuvant trials have failed to demonstrate significant survival advantage in gastric cancer. U.S. Intergroup study (INT-0116) was the first to demonstrate that combined chemoradiation following complete gastric resection improves median relapse-free survival (30 vs. 19 months, p < 0.0001) and overall survival (OS) (36 vs. 27 months, p = 0.01) [4]. The 3-year survival rates were 41% and 50%, respectively (p = 0.005). Following these results, postoperative adjuvant chemoradiation as per the INT-0116 trial, the so-called "Macdonald regimen", became the new standard of care. However, much concern remains regarding the toxicity of the regimen. Forty-one percent of patients in INT-0116 had grade 3 toxicity and 32% had grade 4 toxicity. Three patients (1%) suffered toxic deaths and 31% did not complete treatment due to toxicity.

The aim of this retrospective multi-institutional study was to evaluate the safety and tolerability of the INT-0116 regimen outside the frame of a clinical trial, in a routine clinical practice setting in Israel.

## Patients and Methods

### Patients

The study population consisted of all consecutive patients who were treated by the INT-0116 regimen in one of the participating centers, after the adoption of this regimen as the standard of care, and who fulfilled the study's eligibility criteria. Patients were required to have histologically confirmed adenocarcinoma of the stomach, with macroscopic complete resection of the tumor, disease stage IB to IV (M0) according to the 1997 staging criteria of the American Joint Commission on Cancer [5], an Eastern Cooperative Oncology Group performance status (PS) of ≤ 2, adequate organ function (including cardiac, hepatic and renal functions), adequate bone marrow function (hemoglobin ≥ 10 g/dl; leukocyte count ≥ 4,000/μl; platelet count ≥ 100,000/μl) and an oral caloric intake ≥ 1,500 kcal per day. All patients underwent chest radiographs and abdominopelvic computed tomography to exclude distant metastases.

### Surgery

The surgical requirements for eligibility were surgery with curative intent and *en bloc *resection of the tumor with macroscopically negative margins. As the primary endpoint of the study was safety and we did not expect a difference in that endpoint between patients with microscopic positive margins (R1 resection) and those who underwent complete resection (R0), both groups were included. Eighty-five percent of the patients underwent D0 lymph node dissection and the remaining 15% underwent D1 dissection.

### Chemoradiotherapy

The regimen of fluorouracil (5-FU) and leucovorin (LV) was given according to the INT-0116 trial. Chemotherapy with 5-FU 425 mg/m^2^/day and LV 20 mg/m^2^/day was administered on days 1-5 and was followed by chemoradiotherapy 4 weeks after the start of the initial cycle of chemotherapy. Chemoradiotherapy consisted of 45 Gy of radiation at 1.8 Gy/day, 5 days/week for 5 weeks, with a reduced dose of 5-FU (400 mg/m^2^) plus LV on the first 4 and the last 3 days of radiation. Four weeks after the completion of radiotherapy, two five-day cycles of 5-FU (425 mg/m^2^) and LV were given 4 weeks apart. Radiotherapy was delivered to the tumor bed, as defined by preoperative imaging, the regional lymph nodes, and 2 cm beyond the proximal and distal margins of resection. The dose was prescribed to the isodose line encompassing 95% of the planning tumor volume (PTV).

### Patient evaluation

Patients were followed at 3-month intervals for 2 years, at 6-month intervals for the next 3 years and yearly thereafter. Follow-up consisted of physical examination, complete blood count and liver function tests. Imaging studies and gastroscopy were done when clinically indicated. The site of relapse was classified as follows: locoregional if the tumor was detected within the radiation field (including surgical anastomosis, remnant stomach or gastric bed); peritoneal if the tumor was detected in the peritoneal cavity; and distant in case of liver metastasis or metastases outside the peritoneal cavity.

### Statistical analysis

OS was defined as the time from surgery to death or the last date the patient was known to be alive. Disease-free survival (DFS) was defined as the time from surgery to recurrence of cancer or to the last date the patient was known to be disease-free. The Kaplan-Meier product-limit method [6] was used to estimate survival rates. Comparison of OS and DFS between R0 and R1 patients was performed using the log-rank test. The study was approved by the institutional ethics committee.

## Results

### Patient characteristics

Between 6/2000 and 12/2007, 166 patients with locally advanced gastric cancer received post-operative chemoradiation as per INT-0116 at the participating centers. The patients' characteristics are shown in Table 1. The median age was 63 years (range, 23-86) and the majority (60%) were males. Tumor location was equally distributed in the stomach. Most of the patients had advanced localized disease: 77% had T3-4 tumors and 85% had lymph node involvement.

**Table 1:** Patient Characteristics at the start of postoperative treatment

	Number of patients (%)
**Age, yrs**	
**Median (range)**	63 (23-86)

**Gender**	
**Male**	100 (60)
**Female**	66 (40)

**R status**	
**R0**	129 (78)
**R1**	37 (22)

**Grade**	
**I-II**	32 (19)
**III-IV**	129 (78)
**Unknown**	5 (3)

**Location**	
**Proximal**	48 (29)
**Body**	55 (33)
**Distal**	60 (36)
**Unknown**	3 (2)

**T Stage**	
**T1-T2**	39 (23)
**T3-T4**	127 (77)

**Lymph node status**	
**N0**	25 (15)
**N1**	80 (48)
**N2**	37 (22)
**N3**	24 (15)

### Treatment

As shown in Table 1, all patients underwent gastrectomy with curative intent, 129 (78%) with R0 resection and 37 (22%) with R1 resection. In total, 57% completed the chemotherapy, 87% completed the radiotherapy and 54% completed the entire chemoradiotherapy protocol. The reason for discontinuation was toxicity in all cases.

### Toxicity

Overall, 46.4% of the patients experienced grade ≥ 3 toxicity. Hematological toxicity of any grade was seen in 51% and non-hematological toxicity of any grade was experienced by 90%. The most common severe hematological toxicities were neutropenia and leukopenia (grade ≥ 3 in 30% and 25% of patients, respectively), with 15% of the patients experiencing at least one episode of neutropenic fever (Table 2). The most common severe non-hematological toxicities were nausea, vomiting and diarrhea, with approximately 10% of the patients experiencing grade ≥ 3 of each of these side effects (Table 3). Three patients (1.8%) died due to treatment-related toxicity: one patient died from sepsis, one from diarrhea and one from neutropenic sepsis complicated with small bowel gangrene. Forty-eight patients (29%) were hospitalized for toxicity.

**Table 2:** Hematological toxicity of postoperative chemoradiation

	Median Nadir (/mm^3^)(range)	% of patients	
		
		Grade ≥ 3	All grades
**Any**		32	51

**WBC**	3,200 (180-10, 280)	25	45

**ANC**	1,700 (0-7, 800)	30	43

**Neutropenic fever**	-	15	-

**PLT**	152,500 (11,000-344,000)	3	4

WBC = white blood cells, ANC = absolute neutrophil count, PLT = platelets.

**Table 3:** Non-hematological toxicities of postoperative chemoradiation

Type of toxic effect	% of patients	
	**Grade ≥ 3**	**All grades**

**Any**	25.3	90

**Nausea**	10	65

**Vomiting**	9	40

**Diarrhea**	10	35

**Stomatitis**	7	34

**Anorexia**	5	44

**Abdominal pain**	4	33

**Esophagitis**^**1**^	4	19

**Fatigue**	6	34

**Dermatological**	0	6

**Hepatic**	0	2

**Radiation pneumonitis**	0	1

^1^Including 2 patients with documented Cytomegalous virus (CMV) esophagitis.

### Survival and relapse

**Figure 1:**
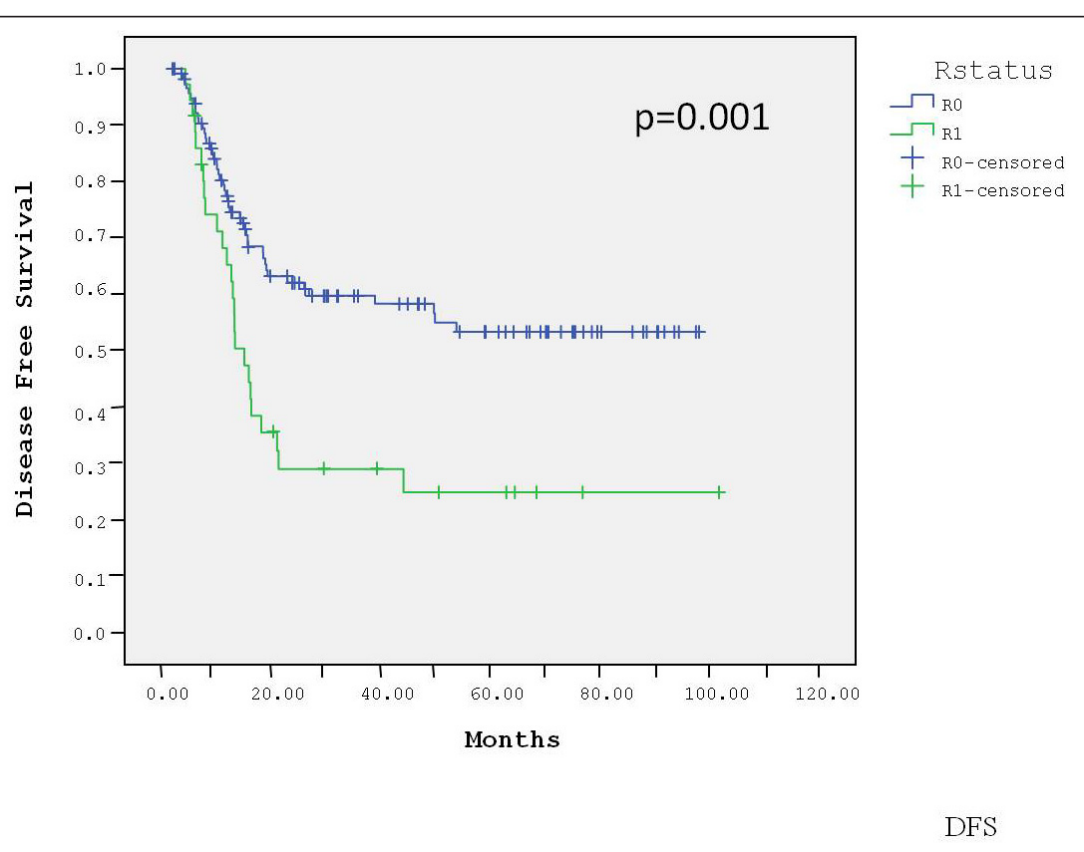
**Disease-free survival by surgical margins**.

**Figure 2:**
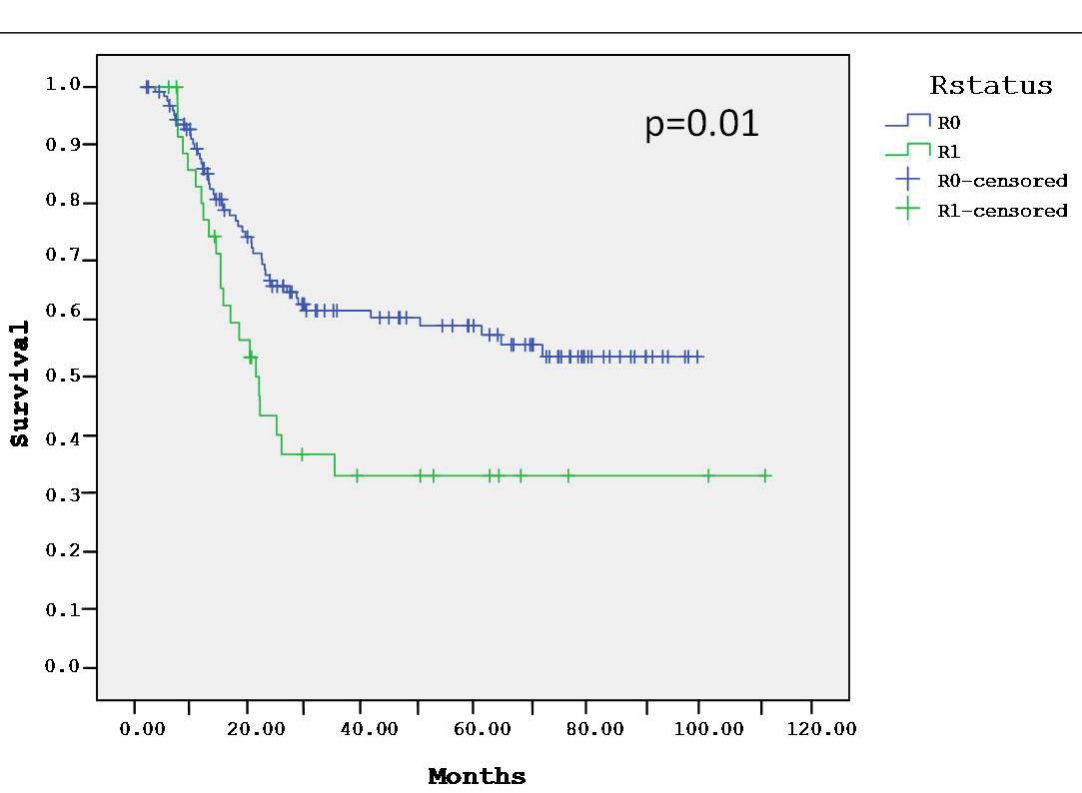
**Overall survival by surgical margins**.

The median follow-up for the entire group was 51 months (range, 2-112). At a median follow-up of 51 months (range, 2-100) for the 129 R0 patients, 38% patients have died of gastric cancer, 61% are alive without evidence of disease and 1% are alive with recurrent disease. Sixty percent of the relapses occurred at distant sites, 22% of them were locoregional and 18% were combined. The estimated 3-year DFS and OS of the R0 patients were 60% and 61%, respectively. The median DFS and OS of these patients have not been reached. With a median follow-up of 51 months (range, 6-112) for the 37 R1 patients, 59% died of gastric cancer, 30% are alive without evidence of recurrence and 11% are alive with disease. Seventy percent of the relapses in this group occurred at distant sites, 15% were locoregional and 15% were combined. The estimated 3-year DFS and OS of the R1 patients were 29% and 33%, respectively. The median DFS in this group was 15 months and the median OS was 22 months. The DFS (p = 0.001) and OS (p = 0.01) were significantly longer in the R0 group compared with the R1 group (Figures 1 and 2). In contrast, there was no difference in outcome between patients who underwent D0 lymph node dissection (85% of patients) and those who underwent D1 dissection (15%) (data not shown).

## Discussion

Adjuvant chemoradiotherapy became a standard treatment option for locally advanced gastric cancer after the publication of the results of the INT-0116 trial that demonstrated OS advantage with this strategy [4]. However, this study is still associated with many open questions and concerns. A key obstacle to the adoption of the chemoradiation used in INT-0116 is the significant toxicity reported for this regimen, including treatment-related deaths. This is of greater concern when such a reportedly toxic regimen is to be administered outside the relatively secured framework of a clinical trial and to be adopted into the routine practice. This multi-institutional Israeli retrospective study was done in this perspective, in order to evaluate the actual performance of the INT-0116 regimen, the so-called "Macdonald regimen", in common daily practice. While the INT-0116 regimen was adopted by most Israeli centers shortly after the original publication, its results have not been reported before.

A comparison of the main patient and tumor characteristics as well as treatment results, in terms of toxicity and efficacy, between the INT-0116 trial and the current study, is depicted in Table 4. The patient populations in both studies were very similar, with a median age in the early 60s and a small male predominance. In both studies most tumors were classified as T3-4 and/or N+ although in the current one there was slightly higher proportion of T3-4 tumors (77% vs. 68%). The toxicity pattern was also very similar, with most toxicities being hematological or gastrointestinal and with comparable rates of severe (grade ≥ 3) toxicities and toxicity-related treatment discontinuations. The rate of hospitalizations was not reported in INT-0116 and was relatively high (32%) in our study. With small absolute numbers in both studies, the rate of toxic deaths in the current study was almost double than in INT-0116 (1.8% vs. 1.0%). To compare the efficacy of chemoradiation in both studies, the DFS and OS rates of the R0 patients in our study were matched with those of the INT-0116 population. We found that the outcome of our patients was at least as good as that of the patients in INT-0116.

**Table 4:** Comparison between the current study and Intergroup-0116

	Current study	INT-0116
	**Patient population**	

**Median age, yrs**	63	60

**Male, %**	60	72

**T3-T4, %**	77	68

**N+, %**	85	85

	**Toxicity**	

**Most common toxicities**	Hem. + GI	Hem. + GI

**Grade 3 toxicity**	35%	41%

**Grade 4 toxicity**	22%	32%

**Hospitalizations**	32%	NA

**Toxic deaths**	**1.8%**	1%

**Discontinuation due to toxicity**	36%	31%

	**efficacy**^1^	

**3-year-DFS**	60%	48%

**3-year-OS**	61%	50%

**Proportion of distant relapses**^**2**^	60%	65%

Hem. = hematological, GI = gastrointestinal, DFS = disease free survival, OS = overall survival.

^1^Efficacy data from the current study is limited only to patients undergoing R0 resection.

^2^Proportion of distant relapses out of all relapses, including those combined with locoregional relapses.

The current study largely confirms the toxicity and efficacy reported in INT-0116. However, several issues seem to deserve attention. First, our higher rate of fatal toxicities is in accordance with the high rate of hospitalizations that we observed, a figure not provided in INT-0116. It is possible that these findings are the result of a less tight monitoring in the common daily practice, but they re-emphasize the toxicity of the regimen and the concerns regarding its place outside a clinical trial framework. Second, the survival rates in our study may be slightly higher than those reported in INT-0116, while our patients had at least as advanced tumors as those in that study. It is unclear whether these are coincidental non-significant differences or whether they actually reflect the improvement in radiotherapy techniques and chemotherapy supportive measures since the original study. Moreover, cross-study comparison is problematic since no randomization or control of potential confounders are feasible. Finally, in both studies the majority of the relapses were distant. This is probably due to the dissimilar effectiveness of the radiotherapy and the chemotherapy used in INT-0116.

INT-0116 study was never repeated. However, during the decade that elapsed since its original publication, multiple other studies were reported on postoperative chemoradiation of gastric cancer. The main features of several representative examples and the INT-0116 study are summarized in Table 5[4,7-22].

**Table 5:** Comparison of different adjuvant chemoradiation studies

First author (country, year)	Type of study	No. of patients	Type of chemotherapy	T3-4	N+	**Any Gr ≥ 3 tox**.	**Gr > 3 Hem. tox**.	**Gr > 3 Non- hem. tox**.	Toxic deaths	Relapse rate	Median survival (months)
MacDonald [4] (USA, 2001)	Phase 3	556	5FU/LV^1^	66%	85%	41%	54%	63%	1%	43%	36

Park [18] (South-Korea,2003)	Phase 2	290	5FU/LV	NA	90%	NA	30%	38%	0%	39%	NA

Hughes [14] (Australia, 2004)	Retrosp.	45^2^	5FU/LV	85%	81%	42%	18%	20%	0%	68%	22.8

Kollmannsberger [20] (Germany, 2005)	Phase 2	86	DDP/5FU PAC/DDP/5FU/LV^4^	NA	NA	NA	81% 89%	56% 56%	0% 0%	19% 33%	NA NA

Kim [9] (South Korea, 2005)	Retrosp.	990	5FU/LV	48%	93%	NA	30%	15%	1%	42%	95

Kassam [15] (Canada, 2006)	Retrosp.	82^2^	5FU/LV^3^ DDP/5FU	53%	82%	56%	33%	34%	0%	32%	NR

Lee [19] (South-Korea, 2006)	Phase 2	31	DDP/5FU	NA	100%	NA	66%	12%	0%	13%	NA

Oechsle [8] (Germany,2007)	Phase 2	157	DDP/PAC/5FU/LV	NA	NA	NA	100%	58%	0.6%^5^	51%	NA
			DDP/5FU/LV				93%	59%		52%	NA
			CPT11/5FU/LV				80%	73%		100%	NA
			DOC/DDP/5FU				100%	30%		30%	NA

Tsang13] (Hong Kong, 2008)	Retrosp.	63^2^	5FU/LV	52%	86%	30%	24%	14%	1.5%	52%	NR

Hofheinz [16] Germany, 2008)	Extend. phase I	32^2^	CAPE/OXALI	44%	97%	NA	21%	42%	0%	47%	NA

Di Costanzo [7] (Italy, 2008)	Phase 3	258	DDP/EPI/5FU/LV^1^	54%	84%	NR	27%	75%	0.8%	48%	57

Leong [22] (Australia, 2009)	Phase 2	54	EPI/DDP/5FU	57%	98%	NA	28%	66%	0%	37%	NR

Aftimos [21] (Lebanon, 2010)	Retrosp.	24	5FU/LV DDP/5FU	75%	71%	NA	20%	36%	0%	22%	75

Chang [17] (Hong Kong, 2011)	Retrosp.	120^2^	5FU/LV	45%	93%	66%	61%	15%	0%	41%	64

Current (Israel, 2011)	Retrosp.	166	5FU/LV	77%	85%	46%	32%	25%	1.8%	30%	NR

Tox. = toxicity, Hem. = hematological, Extend. = extended, Retrosp. = retrospective, DDP = cisplatin, PAC = paclitaxel, EPI = epirubicin, CAP = capecitabine, OX = oxaliplatin, DOC = docetaxel, 5FU = 5 fluorouracil, LV = Leucovorin. NR = not reached, NA = not available.

^1^The regimen used in the treatment arm. The control arm included surgery alone.

^2^These studies included also patients undergoing R1 resection; results of outcome represent patients undergoing R0 resection,

whenever reported separately.

^3^Combined analysis of more than one regimen.

^4^Combined report of consecutive phase II cohorts.

^5^Joint figure for all cohorts combined

Altogether, it is difficult to compare the results of INT-0116 with the other studies, as their data are limited and very heterogeneous, for several reasons. First, with the exception of a single phase III study, by Di Costanzo et al. [7], and a single randomized phase II trial, by Oechsle et al. [8], all studies were phase I or II trials or, more commonly, retrospective analyses. Secondly, aside of the randomized studies described, the retrospective analysis by Kim et al. [9] and the current study, all other studies included only a few dozens of patients each. Thirdly, there was a large variability of the chemoradiation protocol used, including both the chemotherapy regimen and the radiotherapy technique. Lastly, there was significant inconsistency in the endpoints reported, regarding both toxicity and efficacy. Still, review of these studies seems to support the initial perspective of the INT-0116 results, including the appreciation of the toxicity of this treatment as well as its benefit.

To date, the only randomized phase III study to include the INT-0116 regimen is the CALGB 80101 trial. In this trial, patients were randomized to receive the original INT-0116 5FU/LV regimen or ECF (epirubicin/cisplatin/5FU). The chemoradiation regimen was identical in both arms, with continuous infusion of 5FU replacing the bolus 5FU/LV of INT-0116. According to the final results of the study, which have just been reported, ECF is associated with a lower rate of severe toxicities but not with a superior efficacy [10]. Undoubtedly, in light of the toxicity of the INT-0116 regimen and its limited activity, there is an urgent need to improve each one of its components as well as their mode of co-administration. In terms of efficacy, the "Achilles heel" of this regimen is clearly its chemotherapy component. This is evident by the high rate of distant metastases among treated patients. One possible way to improve the efficacy of INT-0116 chemotherapy is to integrate newer chemotherapy agents, such as the taxanes, oxaliplatin and oral fluoropyrimidines, in the treatment. Another promising way is to combine chemotherapy with biological agents. One such potential agent is trastuzumab, which has been shown to improve substantially the results of chemotherapy in advanced gastric cancer with over-expression of HER2 [11]. A different approach to enhance the efficacy of INT-0116 chemotherapy is to modify the timing of its delivery. Perioperative administration of chemotherapy, like in the MAGIC trial [12], is one example for such approach.

As the primary objective of our study was to evaluate the safety of the INT-0116 regimen in daily practice, it included also 37 patients who had microscopic positive (R1) margins. In the absence of clear guidelines on the treatment of such patients, they were given the benefit of doubt that an "adjuvant" treatment might cure their disease or at least postpone its relapse. The effectiveness of this treatment seemed to be limited, as most patients relapsed early, frequently with overt distant spread. Nonetheless, our results seem to imply at least some benefit for the postoperative treatment, as nearly 30% of the R1 patients in our study remained free of recurrence at three years from surgery. Review of the literature reveals very limited data on this relatively common condition. In fact, to our knowledge, our series is by far larger than any of the five previously reported series on patients undergoing R1 gastrectomies [13-17]. In all other series too, except one in which postoperative treatment consisted of the combination of capecitabine and oxaliplatin [16], the INT-0116 regimen was used as adjuvant treatment [13-15,17]. Interestingly, in spite of the very small numbers, all series seem to indicate a similar outcome following R1 resection, with approximately one third of the patients enjoying protracted remissions. The benefit of postoperative chemoradiation in these patients is also suggested by the fact that more than half the patients in our study and others remained free of local recurrence, an unusual figure in the presence of involved margins [13-17]. In the absence of phase III data and consequently lack of clear guidelines in this unfortunately not uncommon situation, our results and earlier ones seem to support the common practice of adding postoperative chemoradiation after R1 gastrectomies.

In summary, the results of the Israeli experience seem to confirm the substantial toxicity and the overall efficacy of postoperative chemoradiation as given in the INT-0116 trial. The mortality rate in our routine practice seems to be higher than in the clinical trial. Altogether, there is a clear need for substantial improvement of the INT-0116 regimen, to reduce its toxicity and enhance its efficacy. In our experience too, involvement of surgical margins is an ominous prognostic sign, even after adjuvant chemoradiation.

## Competing interests

The authors declare that they have no competing interests.

## Authors' contributions

YK: Designed the research, collected the data, analyzed the data and wrote the paper. OP: Designed the research, collected the data, analyzed the data and wrote the paper. EI: Collected the data. KL: Collected the data. SM: Collected the data. SK: Collected the data. NK: Collected the data. RMP: Wrote the paper.

BN: Collected the data. EF: Collected the data and wrote the paper.

AS: Wrote the paper. BB: Designed the research, analyzed the data and wrote the paper. All authors read and approved the final manuscript.
